# External validation of AI-based scoring systems in the ICU: a systematic review and meta-analysis

**DOI:** 10.1186/s12911-024-02830-7

**Published:** 2025-01-06

**Authors:** Patrick Rockenschaub, Ela Marie Akay, Benjamin Gregory Carlisle, Adam Hilbert, Joshua Wendland, Falk Meyer-Eschenbach, Anatol-Fiete Näher, Dietmar Frey, Vince Istvan Madai

**Affiliations:** 1 CLAIM - Charité Lab for AI in Medicine, Charité - Universitätsmedizin Berlin, Berlin, Germany; 2 QUEST Center for Responsible Research, Berlin Institute of Health at Charité Universitätsmedizin Berlin, Berlin, Germany; 3 Institute of Clinical Epidemiology, Public Health, Health Economics, Medical Statistics and Informatics, Medical University of Innsbruck, Innsbruck, Austria; 4 STREAM - Studies of Translation, Ethics and Medicine, School of Population and Global Health, McGill University, Montréal, Canada; 5 Chair for Artificial Intelligence and Formal Methods, Faculty of Computer Science, Ruhr University, Bochum, Germany; 6 Institute of Medical Informatics, Charité - Universitätsmedizin Berlin, Berlin, Germany; 7 Digital Global Public Health, Hasso Plattner Institute for Digital Engineering, University of Potsdam, Potsdam, Germany; 8 Faculty of Computing, Engineering and the Built Environment, School of Computing and Digital Technology, Birmingham City University, Birmingham, UK

**Keywords:** Intensive care unit, Electronic health records, Machine learning, Acute deterioration, External validation

## Abstract

**Background:**

Machine learning (ML) is increasingly used to predict clinical deterioration in intensive care unit (ICU) patients through scoring systems. Although promising, such algorithms often overfit their training cohort and perform worse at new hospitals. Thus, external validation is a critical – but frequently overlooked – step to establish the reliability of predicted risk scores to translate them into clinical practice. We systematically reviewed how regularly external validation of ML-based risk scores is performed and how their performance changed in external data.

**Methods:**

We searched MEDLINE, Web of Science, and arXiv for studies using ML to predict deterioration of ICU patients from routine data. We included primary research published in English before December 2023. We summarised how many studies were externally validated, assessing differences over time, by outcome, and by data source. For validated studies, we evaluated the change in area under the receiver operating characteristic (AUROC) attributable to external validation using linear mixed-effects models.

**Results:**

We included 572 studies, of which 84 (14.7%) were externally validated, increasing to 23.9% by 2023. Validated studies made disproportionate use of open-source data, with two well-known US datasets (MIMIC and eICU) accounting for 83.3% of studies. On average, AUROC was reduced by -0.037 (95% CI -0.052 to -0.027) in external data, with more than 0.05 reduction in 49.5% of studies.

**Discussion:**

External validation, although increasing, remains uncommon. Performance was generally lower in external data, questioning the reliability of some recently proposed ML-based scores. Interpretation of the results was challenged by an overreliance on the same few datasets, implicit differences in case mix, and exclusive use of AUROC.

**Supplementary Information:**

The online version contains supplementary material available at 10.1186/s12911-024-02830-7.

## Introduction

In the intensive care unit (ICU), prognostic scores are used to monitor patients’ severity of illness, predict outcomes, and guide clinical decisions about interventions and resource allocation [1, 2]. These scores have quickly become a fixture in modern critical care and have been adopted in hospitals worldwide [3]. Established scoring systems — such as the Sequential Organ Failure Assessment (SOFA) [4] — rely on a small set of carefully selected parameters to identify patients or patient groups at risk of deterioration [5]. This simplicity comes at the cost of crude prognostication and limited accuracy.

The increasing availability of detailed electronic health records (EHR) has opened the door for developing more sophisticated and personalised scores. Machine learning (ML)-based artificial intelligence (AI) has emerged as a promising tool to leverage the wealth of data [6] and ML-based scores have attracted significant interest within the critical care community [7]. A growing body of literature demonstrates improved accuracy in predicting a diverse range of outcomes including all-cause mortality [8, 9], sepsis [10, 11], kidney injury [12, 13], respiratory failure [14], and more [15, 16].

Despite their promise, ML-based scoring systems are not without risk. Hospitals often differ in the type of patients that they see, the care that they provide, and the systems that they use to document those interactions. One notable challenge in this context is the potential for “overfitting”, where a system’s performance may become overly reliant on unique characteristics of the original patient cohort used for score development. Such overfitting can lead to inaccurate predictions when the system is used in a new clinical environment, where the original unique characteristics are no longer present [6]. Thus, external validation on data from previously unseen hospitals is a critical step in establishing the robustness of these systems and ensuring their reliability across different clinical environments [17, 18]. Unfortunately, external validation is often disregarded in practice [7, 19], raising concerns about the true potential of ML-based scores in the ICU. When a ML-based proprietary score for the detection of sepsis was implemented in clinical practice, an independent evaluation showed that it performed much worse than anticipated [20]. This highlights an emerging translational gap, where theoretical benefits and advertised gains are not realised in clinical practice [18].

This systematic review aimed to address this issue by first determining how frequently external validation is performed in the literature and whether its use has increased in recent years. We then investigated how the performances of ML-based ICU scoring systems typically changed when applied to data from new hospitals. Our results contribute to the ongoing effort of translating reliable ML-based scores to the ICU bedside.

## Methods

### Eligibility criteria

Studies were included in the review if they (1) described the development of an ML-based AI model that (2) provided early warning of acute patient deterioration in (3) ICU settings based on (4) structured, routinely collected EHR data. To be included in the meta-analysis of model performance, models further needed to (5) be externally validated on data from a geographically distinct hospital that was not part of the derivation cohort. Following Shillan et al. (2020) [7], ML was defined as “any form of automated statistical analysis or data science methodology”. Clinical events were considered “acute” if they occurred up to 7 days after the time of prediction. A model gave early warning of such an event if the event was not yet known to the treating clinician at the time of prediction. The ICU was defined as “an area with a sole function to provide advanced monitoring or support to single or multiple body systems” [7]. Models could be externally validated as part of the same publication that developed the model or in a later publication.

Studies were excluded if they: predicted auxiliary outcomes such as length of stay, risk of readmission, laboratory parameters, or values for imputation; used unsupervised learning methods to identify patient subgroups (unless those subgroups were used as input for supervised prediction); included non-ICU patients without providing separate performance metrics (e.g., by including patients from a general ward); required manual note review or prospective data collection of model features; used medical images or natural language processing of free-text notes; only validated the model on data from hospitals that contributed to the development data (including temporal validation on future data); did not report performance in the development dataset.

We included only primary research, excluding reviews and conference abstracts (except for abstracts that were peer-reviewed and paper-length, e.g., from the International Conference on Machine Learning).

### Search strategy

We originally searched the bibliographic databases Ovid MEDLINE and Web of Science for all full-text, peer-reviewed articles matching our search terms published in the English language before April 29th, 2022. Due to delays in the preparation of the manuscript and the fast-moving nature of AI in healthcare, this search was later repeated based on feedback from peers to include articles published before December 13th, 2023. In both cases, we additionally searched the preprint server arXiv for relevant preprints using a custom computer script (see extended supplementary material at 10.17605/OSF.IO/F7J46).

We divided our search into three sub-themes: “Machine Learning and Artificial Intelligence”, “Intensive care setting”, and “Patient deterioration”. Articles were considered for screening if they matched all three themes. Notably, no theme was defined for external validation, which was ascertained manually during screening. Further details of the search strategy including all search terms can be found in the preregistered study Protocol (www.crd.york.ac.uk/prospero, RecordID: 311514). In an attempt to identify models that were validated in a separate, subsequent publication, we further performed a reserve citation search using Dimensions AI (https://www.dimensions.ai/), looking for validation papers that referenced a screened record (see extended supplementary material [21]).

### Study selection

Identified articles were exported from the database as RIS files and imported into the reference management software Zotero (Cooperation for Digital Scholarship; version 6.0.26), where they were deduplicated using Zotero’s semi-automated deduplication tool. Titles and abstracts were independently screened for inclusion by five of the authors (AH, BGC, EMA, JW, PR), with each article being seen by at least two reviewers. For all articles that remained after title and abstract screening, full texts were obtained and independently checked for eligibility by three of the authors (EMA, JW, PR). Before each screening stage, screening was piloted on 25 randomly selected articles. Agreement between authors was assessed using Fleiss’ Kappa [22]. If agreement was found to be unsatisfactory (defined as Kappa < 0.6), decisions were calibrated on another set of 25 articles. If there was uncertainty about the eligibility of an article at any stage of the screening, the article was forwarded to the next stage. Any disagreements were resolved in a consensus meeting. If multiple identified articles describe the same model — e.g., when development and external validation were published in separate articles — the article relating to model validation was included and any missing information on performance in the development dataset was supplemented from the article describing the model development.

### Data collection

Data collection was performed for all included studies, covering information on target outcome(s), data sources, and whether or not the study was externally validated. For the subset of externally validated studies, a more detailed data collection was performed in Numbat Systematic Review Manager [23] using a predefined extraction template (see extended supplementary material [21]). The template was slightly extended prior to data collection to cover all elements defined in the MINimum Information for Medical AI Reporting (MINIMAR) standard [24]. Data collection was performed independently by three authors (original review up to 2022: EMA, PR; update for 2023: JW, PR). We extracted the following information for each validated study: target population; information on the data sources including country of origin, cohort size, outcome prevalence; strategy for dealing with missing data; and performance in internal and external validation. For studies that reported results for more than one algorithm, the performance of the best algorithm during internal validation was recorded. For studies that reported results for more than one outcome, the performance for each outcome was recorded if they were sufficiently different (e.g., mortality and sepsis), otherwise the most acute outcome was chosen (e.g., mortality at 24 h if authors reported both mortality at 24 and 48 h). If a data item could not be ascertained from the main text or supplementary material of the article, it was recorded as missing and no attempt was made to contact study authors for additional data. Additional data items outlined in the protocol (e.g., number of included variables) were extracted but ultimately not used in the analysis; this deviation from the protocol did not affect the overall findings of the review.

### Statistical analysis

Study characteristics and extracted performance metrics were summarised using descriptive statistics and graphical analysis. Changes over time in the proportion of studies performing external validation were assessed using a Chi-square test for linear trend.

Differences in the area under the receiver-operator characteristic curve (AUROC) were analysed using a random-effects model [25]. Parameters were estimated via a Bayesian linear regression model with a single intercept and a normally distributed random effect per study. We used weakly informative normal priors for the mean and half-Cauchy priors for the scale of the random effects [26]. Due to an observed skewed distribution that might unduly influence the results, the difference was modelled with a Cauchy likelihood, which is less sensitive to outliers [27] and is often used for robust regression [28]. Each study’s sample variance was derived using Hanley’s formula [29]. For models estimating mortality — which is a well-defined and well-captured ground truth compared to inferred complications such as sepsis [30] or kidney injury [31] — a sensitivity analysis with an additional fixed effect for mortality was performed. After estimation, we further calculated the proportion of studies in which the absolute difference in AUROC was > ± 0.05. A 0.05 threshold was chosen in line with previous studies [32]. Only studies that reported AUROC performance were included (complete case analysis) and no analysis of heterogeneity between studies or risk of bias was performed.

All analyses were performed in R version 4.2.2 [33]. Bayesian linear models were fitted with Hamiltonian Monte Carlo using the rstan package version 2.21.8 [34]. All results from the database search, screening, full-text review, and data collection as well as the analysis code are available at the Open Science Framework [21]. A study protocol was pre-registered on PROSPERO (www.crd.york.ac.uk/prospero, RecordID: 311514).

## Results

**Fig. 1:**
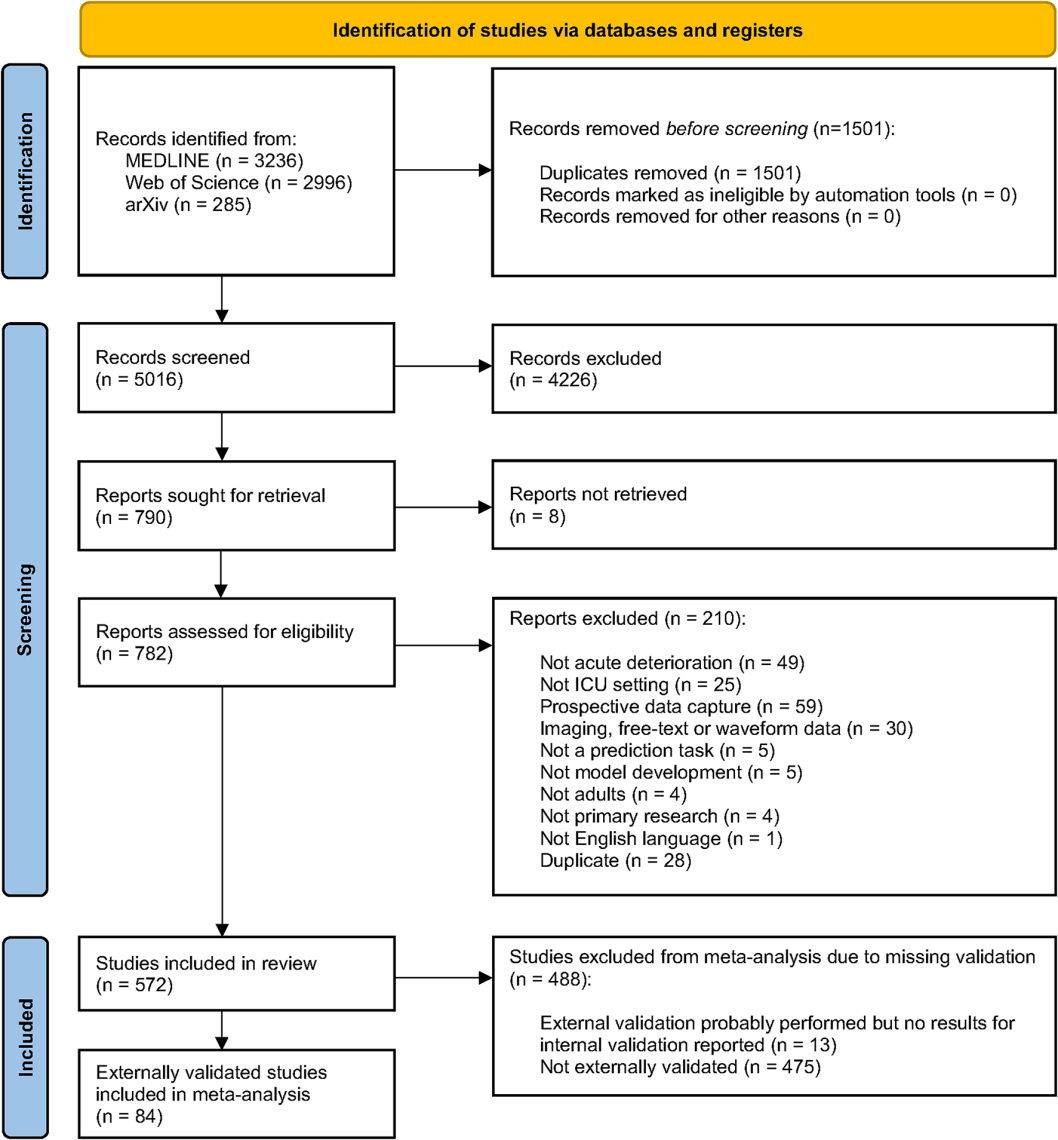
PRISMA flow diagram

We identified 6,517 records in total from MEDLINE (3,236 records), Web of Science (2,996 records), and arXiv (285 records). A detailed flow diagram is shown in Fig. 1. After deduplication, the titles and abstracts of 5,016 records were screened. Full texts were assessed for 782 manuscripts, of which 572 (73.1%) described the prediction of acute deterioration in adult ICU patients from routine data (*included studies*). The main reasons for exclusion were prospective or other non-routine data capture, non-acute outcomes, or the inclusion of image, text, or waveform data (Fig. 1). Of the included studies, 84 (14.7%) were also externally validated (*validated studies;* Supplementary Table 1). No additional validation studies were identified through the reverse citation search (only performed for the original review up to 2022).

### Trend over time

**Fig. 2:**
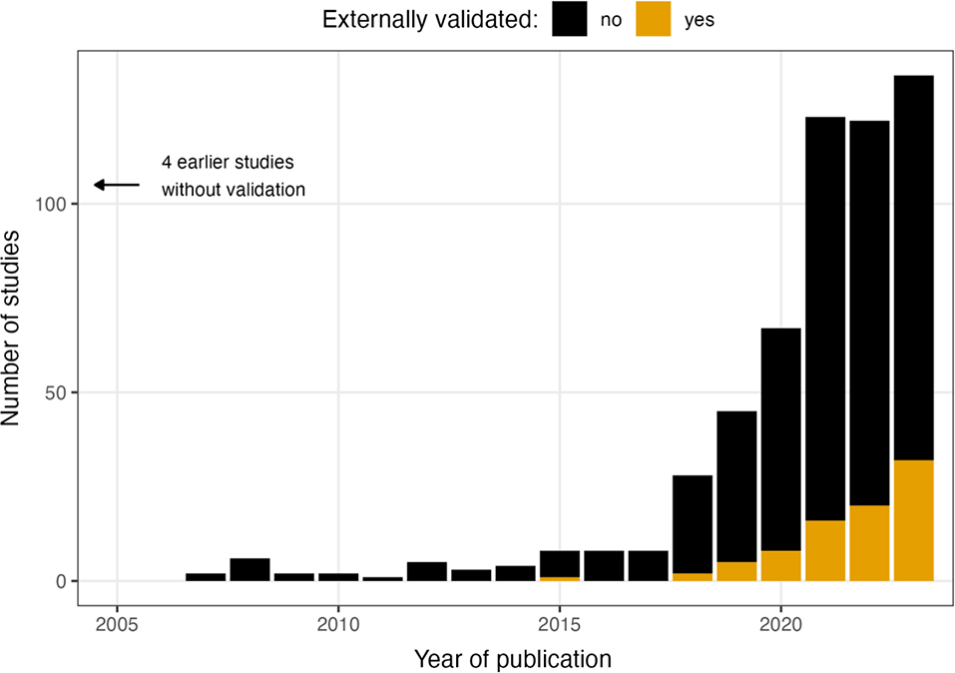
Number of eligible (black) and included externally validated studies (orange) by year of publication

The number of both included and validated studies increased significantly over time (*p* < 0.001) and especially after 2018, with 519 / 572 (90.7%) respectively 83 / 84 (98.8%) studies published in or after that year (Fig. 2). The earliest study performing external validation was published in 2015. Between 2018 and 2022, the proportion of validated studies increased from 2 / 28 (7.1%) to 32 / 134 (23.9%).

### Outcomes

A total of 329 / 572 (57.5%) included studies predicted short-term mortality. The next most commonly predicted outcome was sepsis with 81 / 572 (14.2%), followed by 67 / 572 (11.7%) studies predicting renal complications including acute kidney injury, 36 / 572 (6.3%) studies predicting respiratory complications, and 25 / 572 (4.4%) studies predicting circulatory failure. At 41 / 329 (12.5%), the rate of external validation was slightly lower among studies predicting mortality compared to all studies. If studies predicting mortality were excluded, the proportion of studies that were externally validated — and therefore included in the meta-analysis — notably increased from 14.8% (84 / 572) to 18.5% (50 / 270).

### Sources of data

Externally validated studies overwhelmingly used US data, with 77 / 84 (91.7%) including studies using at least one US dataset for model development or external validation. Chinese data was used in 15 studies, another 15 studies used European data (Netherlands, Switzerland, Denmark, France, Belgium, Italy, Germany), 4 used South Korean data, 2 used Taiwanese data, and 1 each used Israeli, Japanese, and Iranian data.

**Fig. 3:**
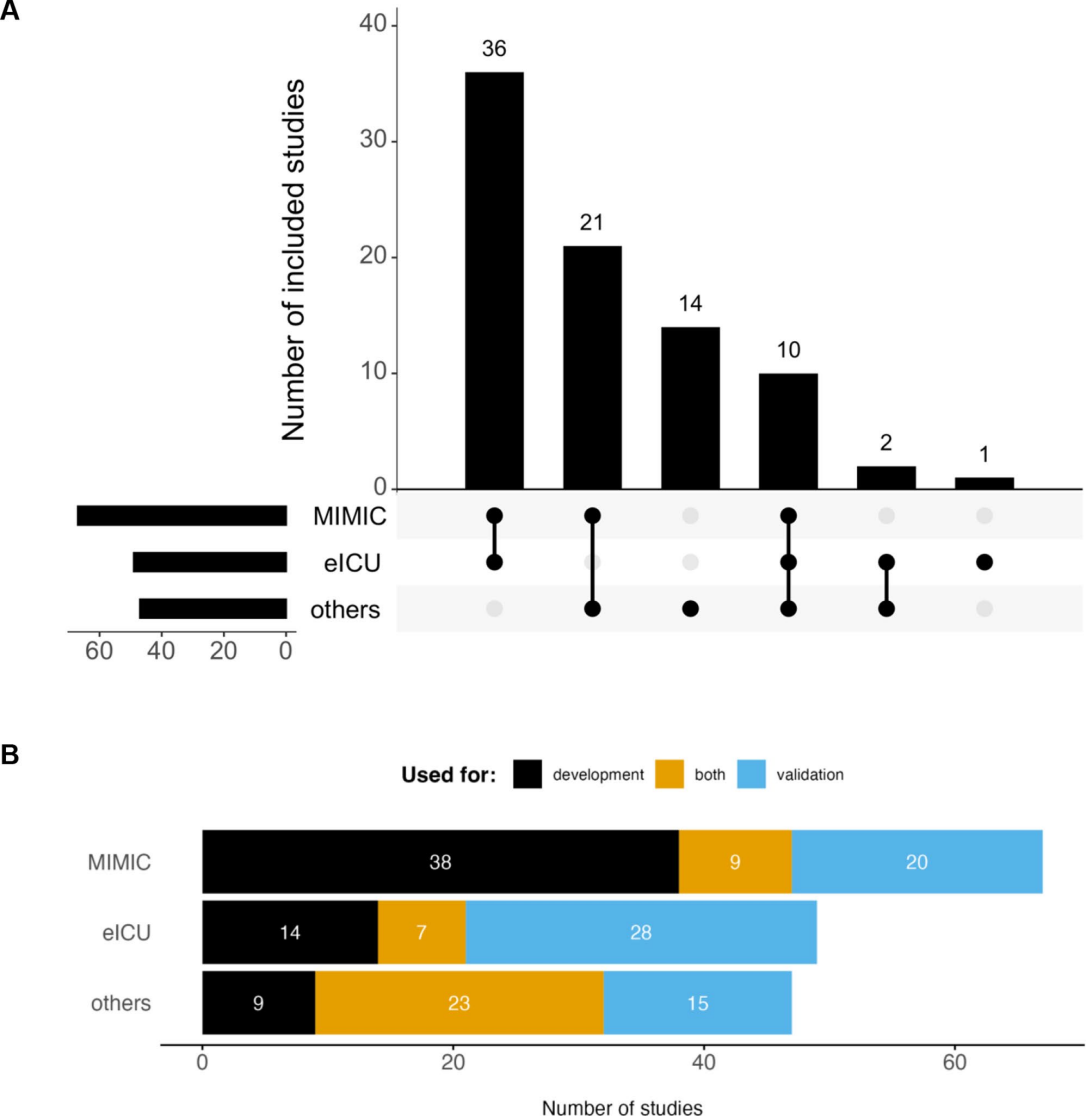
**A** Number of studies that used MIMIC, eICU, and/or one or more other datasets; **B** Number of studies in which MIMIC, eICU, and other datasets were used for model development, external validation, or both

The publicly available datasets MIMIC [35] and eICU [36] were overrepresented among validated studies (Fig. 3 and Supplementary Table 1). MIMIC was used in 67 / 84 (79.8%) of validated studies compared to 344 / 572 (60.1%) of all included studies, with 38 studies using it for model development, 20 for external validation, and 9 for both. eICU was used in 49 / 84 (58.3%) of externally validated studies compared to 119 / 572 (20.8%) of all included studies, 14 times for model development, 28 times for external validation, and 7 times in both capacities. Together, MIMIC and eICU were used in 70 / 84 (83.3%) validated studies, of which they were the only source of data in 36 / 84 (42.9%) studies. AUMCdb [37] and HiRID [15] — two further, more recent public ICU databases — were only used in 7 / 85 (8.2%) and 3 / 85 (3.5%) included studies respectively.

### Performance at new hospitals

**Fig. 4:**
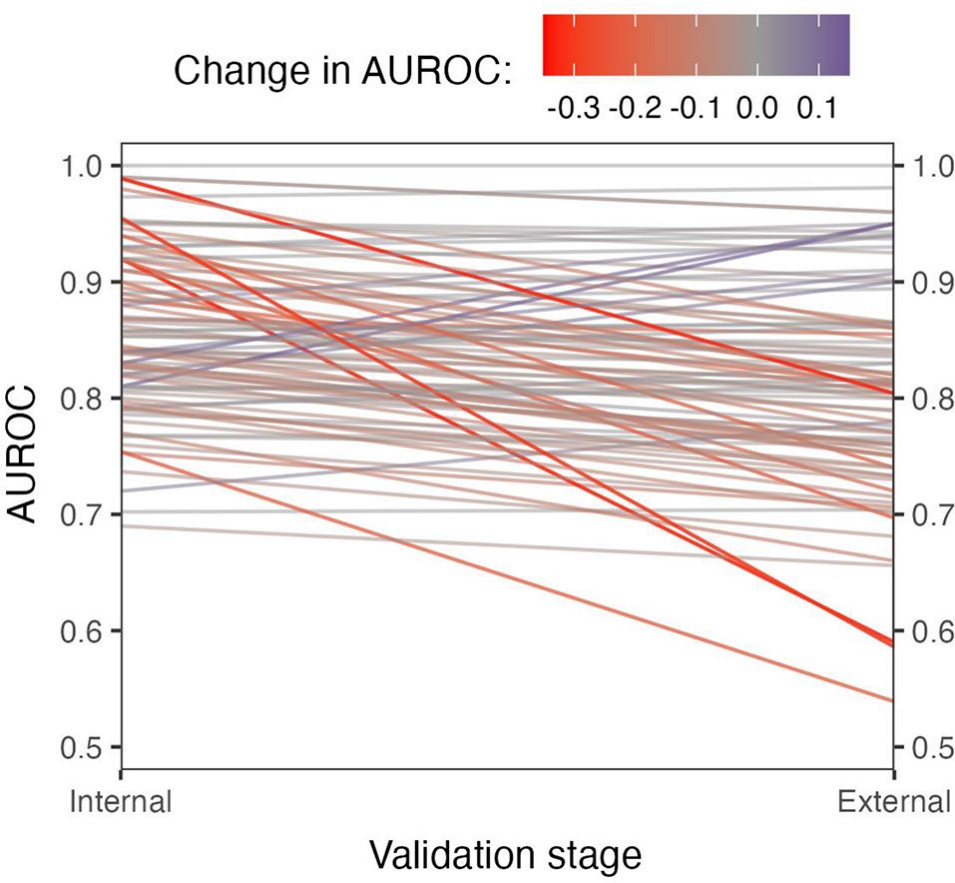
Reported AUROCs for internal and external validation among (*N* = 84 − 2) included studies. Two studies were omitted because they did not report AUROC

All but two of the 84 validated studies reported AUROC. After accounting for sampling variability, model performance in the external validation data was on average − 0.037 (95% credible interval [CI] -0.052 to -0.027) lower than estimated in the internal validation data (Fig. 4). This constitutes a relative decrease of 7–23% in performance, with decreases of up to and more than 50% in some cases. Changes in performance ranged from a maximum increase of 0.140 to a decrease of -0.391 and were much more variable than explained by chance (Supplementary Fig. 1), reflecting the likely substantial heterogeneity introduced by averaging over highly disparate clinical outcomes and underlying patient populations. However, there was no obvious publication bias. In 49.5% of cases, performance loss was < -0.05. On the other end of the spectrum, performance *increased* by > 0.05 in 5.5% of cases — indicating differences in patient populations between train and evaluation cohorts.

There was no evidence for differences between studies predicting death and those that predicted other outcomes (mean difference − 0.003, 95% CI -0.025 to 0.019). We were unable to reliably calculate differences in generalisability by data source, as only 14 / 84 (83.3%) did not use MIMIC or ICU, or due to handling of missing data, as only 17 / 84 studies (14.3%) used multiple imputation. All other studies used model-based single imputation (7 / 84; 8.3%) or unconditional imputation such as last-observation-carried-forward or zero-imputation (40 / 84; 47.6%). Notably, 22 / 84 (26.2%) of studies did not specify how they dealt with missing data.

Commonly reported performance metrics besides AUROC included sensitivity (55 / 84; 65.4%), specificity (52 / 84; 61.9%), accuracy (47 / 84; 60.0%), positive predictive value (45 / 84; 53.6%), and F1 score (39 / 84; 46.4%), although they were reported at a lower rate than AUROC.

## Discussion

This systematic review examined the generalisation of complex, ML-based ICU scoring systems to new hospitals. We considered any score that supports ICU staff through the prediction of imminent patient deterioration from routinely collected EHR data. Leveraging EHR data in this way to improve critical care continues to attract significant research interest, as evidenced by a steady increase in research output. Yet, translating this research into widespread clinical practice — and eventually converting it into patient benefit — requires comprehensive validation of findings, including an evaluation of the scores’ performance at new hospitals. We found that such external validation is still relatively uncommon. Where validation was performed, performance at the new hospital tended to be lower than in the training cohort, often notably so.

### Implications for the translation of AI into clinical practice

Fueled by recent advances in natural language processing and their successful translation to consumer products, there is a reinvigorated hype around the implementation of AI in healthcare [38]. Yet, while many preliminary results keep making the headlines, the proof is in the pudding: a large majority of published results are exploratory in nature, providing only proof-of-concepts [39]. There is a continued lack of verification and clinical validation, blocking the translation of these proof-of-concepts to actual products [18]. In our review, we demonstrate that the issue of inadequate verification extends to ML-based scoring systems: the rate of retrospective external validation — a crucial step to establish validity and robustness — remains low. Less than 20% of identified studies that proposed new scoring systems for the ICU underwent external validation. External validation in this context is an essential step for widespread clinical adoption. Unless a model is solely built for use in the hospital(s) it was developed at — a rare desideratum — it should be judged by its accuracy across a range of hospitals, all of which may potentially use the model in the future. When evaluated this way, we found that average model accuracy as measured by the AUROC decreased by 7–23%. Many ostensibly well-performing scores may thus no longer be suitable for use at the new hospital, a fact that would (and does) go unnoticed in the absence of external validation. To actually facilitate translation to the clinical setting, rigorous external validation must become the standard in most cases when developing ML-based scoring systems and clinical AI more generally. Retrospective external validations in particular aid the early identification of model deficiencies, highlighting the need for training on a broader variety of training data [40] or performing local model updates prior to deployment. While there is still a long way to go to make such external validation the default, our review at least suggests that there is a growing recognition of its importance among researchers: over 80% of all identified studies performing external validation were published in 2018 or later.

### Interpretation of external validation results

The infrequent external validation of ML models for the prediction of acute events in the ICU was already noted in a 2019 systematic review, with only 7% of studies at the time using geographically independent data for model validation [7]. This has been echoed in more recent, disease-specific reviews looking at models for sepsis [19] and acute kidney injury [41]. While we showed that this percentage has somewhat improved since, we also find that challenges remain even if external validation is performed.

While we observed a tendency for reduced model performance in external data, the magnitude of reduction was milder than anticipated from previous studies [40, 42–44]. This may partially be explained by the performance metric. We focused on the AUROC as the primary effect measure since it allowed performing a meta-analysis due to its popularity and its comparability across different levels of prevalence. However, AUROC may be less sensitive to changes in the data. For example, while the drop in AUROC in the PhysioNet CinC challenge 2019 [42] was generally mild and in line with our findings, the “utility of prediction” — a custom metric defined as a timely prediction within 12 h before to 3 h after the onset of sepsis — in the new hospital was worse than not predicting at all. The average reduction in performance might have been more pronounced if another metric such as utility or normalised AUPRC were used instead of AUROC. Unfortunately, it was not possible to include such metrics in a meta-analysis due to their infrequent reporting. We recommend that future validation studies systematically report multiple performance metrics that represent the performance holistically.

The observed moderate reduction in average performance may have also been driven by the non-negligible number of models whose performance *increased* during external validation. Whereas minor fluctuations may occur due to sampling variability, a model’s performance shouldn’t notably increase in the external validation cohort. If it does, this suggests that there may be systematic differences in case mix between the training and validation cohorts — rendering the performances incomparable. If cohorts cannot be defined well enough to ensure their comparability, we recommend also reporting the performance of a model trained solely on the validation data. This provides a (potentially overfit) upper limit on what might have been achieved in the external data [40] and thus allows readers to take any distorting effects of case mix into consideration.

### Barriers to meaningful external validation

Although the rate of external validation is slowly rising, it appears almost exclusively confined to a few open-source validation sets, most prominently MIMIC [35] and eICU [36]. A version of MIMIC was used in almost 80% of all identified studies that performed external validation. This is potentially problematic, as studies worldwide are thus largely judged by their ability to retain performance in patients from the single US hospital included in MIMIC, which very likely does not represent the wider ICU population. This means that users and reviewers need to closely scrutinise claims of external validation in the area of ICU scoring systems if they judge tools that are to be used outside of the specific clinical settings captured by MIMIC. This also highlights that while large open-source datasets are able to fuel a large number of publications in certain areas, they do not necessarily by themselves improve the ability to build models that generalize, limiting their impact on successful translation to the clinical setting.

Greater diversity in external validation is hampered by continued difficulties in accessing data from multiple healthcare providers. Concerns about data security and privacy discourage institutions from sharing their data. Even when there is a will to share, differences in EHR systems, issues of data quality, and lack of semantic interoperability often frustrate pooling of data without considerable standardisation and harmonisation efforts. In light of this, it is perhaps not surprising that external validation makes disproportionate use of those few datasets that are readily accessible. To overcome these barriers, more work will be needed to support researchers in accessing diverse, multicentre data, including technologies for secure data access (e.g., federated learning [45, 46]) and tooling that supports data interoperability [47–49].

We further support recent efforts to bring nuance into the discussion around “clinical validation” [50–52]. While external validation on retrospective data is a crucial step in most translation efforts, it is not sufficient to perform *any*, and especially not *a single* external (retrospective) validation to support the claim of generalisability [53, 54]. Nor is it likely that a model will be universally generalisable. Instead, the data used for external validation should be carefully chosen to reflect the model’s anticipated use and specific claims of generalisability. For example, a model developed for use in Germany should be validated in Germany, and may be considered generalisable only if it proves reasonably robust across a range of relevant German institutions. The same model may later be found to perform worse in lower-resource settings, suggesting that — although it did generalise within Germany — it may not generalise to those contexts.

### Strengths and limitations

We used a thorough, pre-defined search strategy to identify all relevant studies, covering two major bibliographic databases as well as the most relevant preprint server for ML research. Inclusion criteria were carefully assessed for all identified records by at least two reviewers, and we additionally performed a reverse reference search to ensure we did not miss validation results that were published as stand-alone manuscripts.

To allow for direct comparability of AUROC in the development and validation data, we limited our analysis to external validation on retrospective, routine data. We did not capture validation that was performed by prospectively collecting additional data or within clinical trials, which may be considered the true test of a clinical prediction model. This has two important implications. First, the proportion of validated studies may be higher than reported here, especially in the years preceding the availability of large open-source datasets. Second, the reported performances do not necessarily imply clinical usability but rather reflect the stability of study results across different sets of data. Nevertheless, external validation in retrospective data is an invaluable step to assess the usability of a prediction model in clinical practice and should be considered for any study developing prediction models from routine data. Existing findings are fundamental to the conception of future studies and basing future research on ‘false’ or non-robust results can significantly hinder genuine innovation in the field, creating a substantial drain on both time and financial resources.

Due to the anticipated heterogeneity of studies, we limited ourselves to a descriptive summary of study results and trends. We did not perform a risk of bias assessment. Previous studies that assessed study quality reported a neglect of model calibration, inappropriate internal validation, and overall lack of reproducibility [19, 41], all of which may also have been presented in the studies included here. Our results also assume that there were no systematic differences between studies that did and did not get externally validated. This is a strong assumption. For example, studies that were externally validated may be more generalisable to begin with because good performance in new dataset(s) was an explicit part of the study objectives. In this case, the true performance drop among non-validated studies may be even greater than estimated here.

## Conclusion

The increasing availability of routine data capture and open-source ICU data sources are gradually removing the barriers to routine external validation of ML-based scoring systems. External validation can provide invaluable information on the robustness of newly proposed scores and their potential for widespread adoption. However, while some external validation is certainly better than none, our results caution against choosing datasets for external validation solely based on their ease of access. Results derived from external validation efforts will only be truly useful if the data used for validation is carefully selected to reflect the model’s intended use, taking into account shifts in data quality, patient case mix, and any other factors that may impact model performance. This will require concerted efforts to facilitate access to more diverse, multicentre data as well as a systematic reporting of a range of performance metrics to allow for a more meaningful assessment of model performance.

## Electronic supplementary material

Below is the link to the electronic supplementary material.

Supplementary Material 1

## Data Availability

The datasets generated and/or analysed during the current study are available in the OSF repository, 10.17605/OSF.IO/F7J46.

## Abbreviations

AI: Artificial Intelligence
AUROC: Area Under the Receiver-Operator Characteristic
EHR: Electronic Health Records
ICU: Intensive Care Unit
ML: Machine Learning
SOFA: Sequential Organ Failure Assessment
