# Supplementary material for: External validation of AI-based scoring systems in the ICU: a systematic review and meta-analysis
Source: BMC Med Inform Decis Mak. 2025 Jan 6;25:5. doi: 10.1186/s12911-024-02830-7 (PMC11702098; doi:10.1186/s12911-024-02830-7)
Supplement: Supplementary file 1 — Supplementary Material 1 [file 12911_2024_2830_MOESM1_ESM.docx]

**Supplementary Table 1** - Summary of externally validated studies

| **Authors** | **Year** | **Cohort** | **Outcome** | **Data source** | | | **Sample size** | | **AUROC** | |
| --- | --- | --- | --- | --- | --- | --- | --- | --- | --- | --- |
|  |  |  |  | Dev | Val |  | Dev | Val | Dev | Val |
| Pirracchio [[1]](https://paperpile.com/c/SmPg0w/JUxnd) | 2015 | All patients* | Mortality | MIMIC | Other (France) |  | 25k | 200- | .88 | .94 |
| Delahanty [[2]](https://paperpile.com/c/SmPg0w/FJNMl) | 2018 | All patients* | Mortality | Other (US) | Other (US) | ⭐ | 147k | 90k | .95 | .94 |
| Moon [[3]](https://paperpile.com/c/SmPg0w/bSi45) | 2018 | All patients* | Neurological | Other (Korea) | Other (Korea) | ⭐ | 3k | 325- | .90 | .72 |
| Huang [[4]](https://paperpile.com/c/SmPg0w/Qk03K) | 2019 | All patients* | Mortality | eICU | ^+,‡^ |  | 28k | ‡ | .74 | .68 |
| Liu [[5]](https://paperpile.com/c/SmPg0w/VeWLP) | 2019 | Sepsis | Sepsis (shock) | MIMIC | eICU |  | 15k | ? | .93 | .85 |
| Shickel [[6]](https://paperpile.com/c/SmPg0w/IZM9Y) | 2019 | All patients* | Mortality | Other (US)  MIMIC | ^+^ |  | 36k  49k | ^+^ | .91  .91 | .90  .90 |
| van Wyk [[7]](https://paperpile.com/c/SmPg0w/sOF9a) | 2019 | All patients* | Sepsis | Other (US) | ^+^ | ⭐ | 586- | ^+^ | - | - |
| Nielsen [[8]](https://paperpile.com/c/SmPg0w/zhudH) | 2019 | All patients* | Mortality | Other (DK) | Other (DK) | ⭐ | 10k | 2k | .79 | .73 |
| Kang [[9]](https://paperpile.com/c/SmPg0w/yBcLH) | 2020 | All patients* | Mortality | MIMIC  eICU | ^+^ |  | 21k  198k | ^+^ | .90  .87 | .86  .72 |
| Zhao [[10]](https://paperpile.com/c/SmPg0w/ePSdx) | 2020 | Sepsis | Other | MIMIC | eICU |  | 11k | 35k | .87 | .84 |
| Roimi [[11]](https://paperpile.com/c/SmPg0w/Eqj9R) | 2020 | Susp. bacteraemia | Infection | MIMIC  Other (Israel) | ^+^ |  | 2k  1k | ^+^ | .89  .92 | .59  .60 |
| Hyland [[12]](https://paperpile.com/c/SmPg0w/SZeNB) | 2020 | All patients* | Circulatory | HiRID | MIMIC |  | 36k | 9k | .94 | .90 |
| Reyna [[13]](https://paperpile.com/c/SmPg0w/aaEAf) | 2020 | All patients* | Sepsis | MIMIC  Other (US) | Other (US) |  | 20k  20k | ? | .82  .86 | .81 |
| Wang [[14]](https://paperpile.com/c/SmPg0w/U4qzy) | 2020 | All patients* | Renal | Other (China) | MIMIC |  | 11k | 46k | .81 | .95 |
| Liu [[15]](https://paperpile.com/c/SmPg0w/peoPe) | 2020 | MODS | Mortality | MIMIC  eICU | Other (China) |  | 15k  34k | 439- | .86  .85 | .84 |
| Zhou [[16]](https://paperpile.com/c/SmPg0w/gSFDD) | 2020 | Viral pneumonia | Mortality | eICU | MIMIC |  | 4k | 937- | .77 | .66 |
| Rahman [[17]](https://paperpile.com/c/SmPg0w/NJXla) | 2021 | All patients* | Circulatory | eICU | MIMIC |  | 216k | 16k | .82 | .90 |
| Zhi [[18]](https://paperpile.com/c/SmPg0w/rTofq) | 2021 | Sepsis | Mortality | MIMIC | Other (China) |  | 2k | 125- | .75 | .54 |
| Holder[[19]](https://paperpile.com/c/SmPg0w/fZF71) | 2021 | Sepsis | Other | Other (US) | Other (US) | ⭐ | 9k | 5k | .81 | .77 |
| Hur [[20]](https://paperpile.com/c/SmPg0w/LuXki) | 2021 | All patients* | Neurological | Other (Korea) | MIMIC |  | 12k | 2k | .92 | .70 |
| Chen [[21]](https://paperpile.com/c/SmPg0w/Oh6rk) | 2021 | All patients* | Renal | MIMIC | Other (China) |  | 46k | 226- | .83 | .79 |
| He [[22]](https://paperpile.com/c/SmPg0w/dHWb9) | 2021 | Sepsis and AKI | Renal | Other (China) | MIMIC |  | 209- | 509- | 1.0 | 1.0 |
| Shashikumar [[23]](https://paperpile.com/c/SmPg0w/47C1I) | 2021 | All patients* | Sepsis | Other (US) | Other (US) | ⭐ | 17k | 46k | .95 | .93 |
| Levi [[24]](https://paperpile.com/c/SmPg0w/KFvTX) | 2021 | GI bleeding | Other | MIMIC  eICU | ^+^ |  | 4k  10k | ^+^ | .81  .79 | .76  .80 |
| Ding [[25]](https://paperpile.com/c/SmPg0w/kfzwj) | 2021 | Sepsis and AKI | Renal | MIMIC | eICU |  | 7k | 3k | .70 | .70 |
| Huang [[26]](https://paperpile.com/c/SmPg0w/Bik2z) | 2021 | AKI | Mortality | MIMIC | eICU |  | 4k | 1k- | .91 | .82 |
| Singhal [[27]](https://paperpile.com/c/SmPg0w/AJ8dZ) | 2021 | COVID-19 | Respiratory | Other (US) | Other (US) | ⭐ | 6k | 611- 77- | .90 | .85  .88 |
| Sung [[28]](https://paperpile.com/c/SmPg0w/jEVPG) | 2021 | All patients* | Mortality | Other (Korea) | Other (Korea) | ⭐ | 22k | 2k | .99  .77  .84 | .96  .77  .80 |
| Liu [[29]](https://paperpile.com/c/SmPg0w/IQd11) | 2021 | All patients* | Sepsis | Other (US) | eICU |  | 882- | 6k | .72 | .78 |
| Shawwa [[30]](https://paperpile.com/c/SmPg0w/K2qw8) | 2021 | All patients* | Renal | Other (US) | MIMIC |  | 98k | 19k | .69 | .66 |
| Mamandipoor [[31]](https://paperpile.com/c/SmPg0w/5j1YE) | 2021 | All patients* | Other | eICU | MIMIC |  | 17k | 13k | .84 | .83 |
| Moor [[32]](https://paperpile.com/c/SmPg0w/liFQe) | 2021 | All patients* | Sepsis | MIMIC  eICU  HiRID  AUMCdb | ^+^ |  | 37k  57k  27k  16k | ^+^ | .83  .80  .83  .92 | .71  .75  .73  .81 |
| Peng [[33]](https://paperpile.com/c/SmPg0w/lzFYg) | 2022 | HF | Renal | MIMIC | eICU |  | 9k | 10k | .81 | .82 |
| Luo [[34]](https://paperpile.com/c/SmPg0w/IvBY1) | 2022 | HF | Mortality | MIMIC | eICU |  | 6k | 1k | .83 | .81 |
| Kim [[35]](https://paperpile.com/c/SmPg0w/mgnH6) | 2022 | Cardiac arrest and MV | Mortality | eICU | MIMIC |  | 2k | 86- | .83 | .76 |
| Fu [[36]](https://paperpile.com/c/SmPg0w/s0TsG) | 2022 | Circulatory failure | Renal | MIMIC | eICU |  | 1k | 1k | .82 | .73 |
| Zhang [[37]](https://paperpile.com/c/SmPg0w/8IdMW) | 2022 | Cerebrovascular disease | Renal | MIMIC | Other (China) |  | 3k | 499- | .88 | .78 |
| Jiang [[38]](https://paperpile.com/c/SmPg0w/wGbal) | 2022 | Sepsis | Other | Other (China) | MIMIC |  | 1k | 688- | .89 | .77 |
| Liang [[39]](https://paperpile.com/c/SmPg0w/URq6N) | 2022 | All patients* | Renal | Other (China) MIMIC | AUMCdb |  | 6k  37k | 15k | .86  .86 | .87 |
| Sharafutdinov [[40]](https://paperpile.com/c/SmPg0w/c9IdC) | 2022 | All patients* | Respiratory | Other (Germany)  MIMIC | + |  | 13k  3k  1k  8k | + | .83  .94  .91  .79 | .91  .90  .91  .81 |
| Chang [[41]](https://paperpile.com/c/SmPg0w/KW0cg) | 2022 | RRT | Mortality | MIMIC | eICU |  | 1k | 2k | .79 | 0.81 |
| Bai [[42]](https://paperpile.com/c/SmPg0w/9bIJP) | 2022 | Sepsis | Respiratory | eICU | MIMIC |  | 19k | 12k | .90 | 0.80 |
| Kang [[43]](https://paperpile.com/c/SmPg0w/FeXqK) | 2022 | All patients* | Bleeding | MIMIC | eICU |  | 6k | 220 | .94 | .74 |
| Rangan [[44]](https://paperpile.com/c/SmPg0w/SiWHy) | 2022 | All patients* | Sepsis | Physionet Challenge 2019 (US) | Physionet Challenge 2019 (US) | ⭐ | 3k | 10k | .93 | .94 |
| Luo [[45]](https://paperpile.com/c/SmPg0w/gJLEv) | 2022 | Sepsis and AKI | Mortality | MIMIC | eICU |  | 12k | 4k | .85 | .85 |
| Chen [[46]](https://paperpile.com/c/SmPg0w/G0hNq) | 2022 | All patients* | Sepsis | MIMIC | Other (China) |  | 7k | 453 | .98 | .86 |
| Alfieri [[47]](https://paperpile.com/c/SmPg0w/GesrY) | 2022 | All patients* | Renal | MIMIC  eICU | AUMCdb |  | 36k | 11k | .89 | .91 |
| Choi [[48]](https://paperpile.com/c/SmPg0w/yFJ9k) | 2022 | All patients* | Mortality | Other (South Korea) | + | ⭐ | 62k  24k | + | .98  .96 | .59  .65 |
| Yang [[49]](https://paperpile.com/c/SmPg0w/uuvzt) | 2022 | HF with diabetes | Mortality | MIMIC | eICU |  | 3k | 4k | .83 | .75 |
| Dung-Hung [[50]](https://paperpile.com/c/SmPg0w/Ttx8r) | 2022 | All patients* | Circulatory | eICU | Other (Taiwan) |  | 216k | 17k | .82 | .76 |
| Mirzakhani [[51]](https://paperpile.com/c/SmPg0w/VTf3q) | 2022 | All patients* | Mortality | Other (Iran) | Other (Iran) | ⭐ | 840 | 150 | .84 | .79 |
| Wu [[52]](https://paperpile.com/c/SmPg0w/rFln1) | 2022 | Inhalation-induced ARDS | Respiratory | eICU | MIMIC  Other (China) |  | 5 | 3  15 | .84 | .90  .91 |
| Rockenschaub [[53]](https://paperpile.com/c/SmPg0w/9lSvh) | 2023 | All patients* | Mortality, Renal, Sepsis § | MIMIC  eICU  HiRID  AUMCdb | + |  | 52k  113k  13k  11k | + | .86  .84  .84  .84 | .83  .82  .76  .77 |
| Mesinovic [[54]](https://paperpile.com/c/SmPg0w/BKWtg) | 2023 | MI | Mortality | eICU | MIMIC |  | 26k | 1k | .86 | .80 |
| Contreras [[55]](https://paperpile.com/c/SmPg0w/zTXVd) | 2023 | All patients* | Mortality | eICU  Other (US) | ‡  MIMIC |  | 137k | 102k | .93 | .95 |
| Zhu [[56]](https://paperpile.com/c/SmPg0w/J307m) | 2023 | All patients* | Mortality | MIMIC | eICU |  | 30k | 109k | .86 | .86 |
| Zhang [[57]](https://paperpile.com/c/SmPg0w/2Rtp8) | 2023 | Infection | Sepsis | MIMIC | Other (China) |  | 4k | 507 | .83 | .95 |
| Liu [[58]](https://paperpile.com/c/SmPg0w/CJlSB) | 2023 | Age > 65 years | Mortality | MIMIC | eICU  AUMCdb |  | 50k | 22k  3k | .87 | .84  .83 |
| Ren [[59]](https://paperpile.com/c/SmPg0w/j9geg) | 2023 | Burns | Neurological | Other (China) | Other (China) | ⭐ | 518 | 118 | .96 | - |
| Wang [[60]](https://paperpile.com/c/SmPg0w/Ze31B) | 2023 | Pneumonia | Mortality | MIMIC | eICU |  | 5k | 14k | .76 | .71 |
| Alfieri [[61]](https://paperpile.com/c/SmPg0w/MnmHf) | 2023 | All patients* | Renal | MIMIC  AUMCdb | eICU  Other (Italy) |  | 9k | 7k  1k | .89 | .88  .91 |
| Shi [[62]](https://paperpile.com/c/SmPg0w/OiimZ) | 2023 | GI bleeding | Renal | eICU | MIMIC |  | 10k | 3k | .87 | .84 |
| Huang [[63]](https://paperpile.com/c/SmPg0w/5KJZE) | 2023 | All patients* | Renal | Other (Taiwan) | Other (Taiwan) | ⭐ | 17k | 12k  12k  11k  3k | .93 | .87  .81  .83  .76 |
| Zeng [[64]](https://paperpile.com/c/SmPg0w/Vcm6q) | 2023 | All patients* | Mortality | MIMIC | eICU |  | 66k | 151k | .92 | .86 |
| Zheng [[65]](https://paperpile.com/c/SmPg0w/7cBjM) | 2023 | Liver cirrhosis | Renal | eICU | MIMIC |  | 1k | 789 | .81 | .77 |
| Ren [[66]](https://paperpile.com/c/SmPg0w/nRUbU) | 2023 | Acute pancreatitis | Mortality | MIMIC | eICU |  | 856 | 425 | .84 | .86 |
| Ma [[67]](https://paperpile.com/c/SmPg0w/tijgM) | 2023 | All patients* | Other | MIMIC | eICU |  | 4k | 3k | .97 | .98 |
| Fan [[68]](https://paperpile.com/c/SmPg0w/kyZvg) | 2023 | Sepsis and AKI | Mortality | MIMIC | Other (China) |  | 2k | 100 | .91 | .81 |
| Yamga [[69]](https://paperpile.com/c/SmPg0w/JxbFv) | 2023 | Circulatory failure | Mortality | eICU | MIMIC |  | 9k | 2k | .83 | .76 |
| Neyra [[70]](https://paperpile.com/c/SmPg0w/nmpiy) | 2023 | AKI | Mortality, Renal § | Other (US) | Other (US) | ⭐ | 7k | 2k | .79 | .74 |
| Zheng [[71]](https://paperpile.com/c/SmPg0w/22kRw) | 2023 | Liver cirrhosis | Mortality | MIMIC | eICU |  | 788 | 501 | .77 | .76 |
| Bao [[72]](https://paperpile.com/c/SmPg0w/5xAYe) | 2023 | Sepsis | Mortality | MIMIC | eICU |  | 13k | 9k | .99 | .96 |
| Huang [[73]](https://paperpile.com/c/SmPg0w/27maF) | 2023 | MV | Renal | MIMIC | eICU |  | 5k | 1k | .81 | .80 |
| Verhaeghe [[74]](https://paperpile.com/c/SmPg0w/f2Pyy) | 2023 | All patients* | Cardiological | AUMCdb | MIMIC Other (Belgium) |  | 18k | 59k  23k | .81 | .77  .84 |
| Liao [[75]](https://paperpile.com/c/SmPg0w/hHmqo) | 2023 | All patients* | Mortality, Respiratory, Circulatory failure § | MIMIC  eICU | + |  | 39k  126k | + | .88  .86 | .82  .81 |
| Xie [[76]](https://paperpile.com/c/SmPg0w/A9QwD) | 2023 | Diabetic ketoacidosis | Mortality | MIMIC | eICU |  | 961 | 4k | .95 | .94 |
| Zhou [[77]](https://paperpile.com/c/SmPg0w/G7oVs) | 2023 | Sepsis and AKI | Mortality | MIMIC | Other (China) |  | 16k | 132 | .83 | .75 |
| Ishii [[78]](https://paperpile.com/c/SmPg0w/H2iOw) | 2023 | All patients* | Mortality | Other (Japan) | MIMIC |  | 39k | 30k | .92 | .82 |
| Huang [[79]](https://paperpile.com/c/SmPg0w/DfL7J) | 2023 | Lung cancer | Mortality | MIMIC | eICU |  | 3k | 2k | .92 | .93 |
| Ouyang [[80]](https://paperpile.com/c/SmPg0w/1S5cx) | 2023 | Cerebral infarction | Mortality | eICU | MIMIC |  | 3k | 1k | .80 | .74 |
| Lei [[81]](https://paperpile.com/c/SmPg0w/Ojh8X) | 2023 | Hip fracture | Mortality | MIMIC | eICU |  | 391 | 165 | .80 | .72 |
| Ye [[82]](https://paperpile.com/c/SmPg0w/qxA5r) | 2023 | CKD and CAD | Mortality | MIMIC | eICU |  | 4k | 2k | .95 | .87 |
| Zhuang [[83]](https://paperpile.com/c/SmPg0w/sFyRD) | 2023 | Sepsis | Mortality | MIMIC | eICU  Other (China) |  | 17k | 201k  1k | .84 | .83  .68 |
| Zhang [[84]](https://paperpile.com/c/SmPg0w/NSPwM) | 2023 | Sepsis | Neurological | MIMIC | eICU |  | 15k | 2k | .79 | .70 |

* “All Patients” are defined as a general adult ICU patient population without specifying additional health conditions (e.g., admitted with sepsis). + Datasets were used alternatingly for development and validation. ‡ eICU includes data from 208 different hospitals and may thus be used for both development and validation if split by hospital. § Only results for the first outcome are shown in this table. ⭐ Does not use MIMIC or eICU data.

AKI, acute kidney injury: AUROC, area under the receiver operating characteristic; CAD, coronary artery disease; CKD, chronic kidney disease; HF, heart failure; DK, Denmark; GI, gastro-intestinal; MI, myocardial infarction; MODS, Multi-organ dysfunction syndrome; MV, mechanical ventilation; RRT, renal replacement therapy; US, United States

**
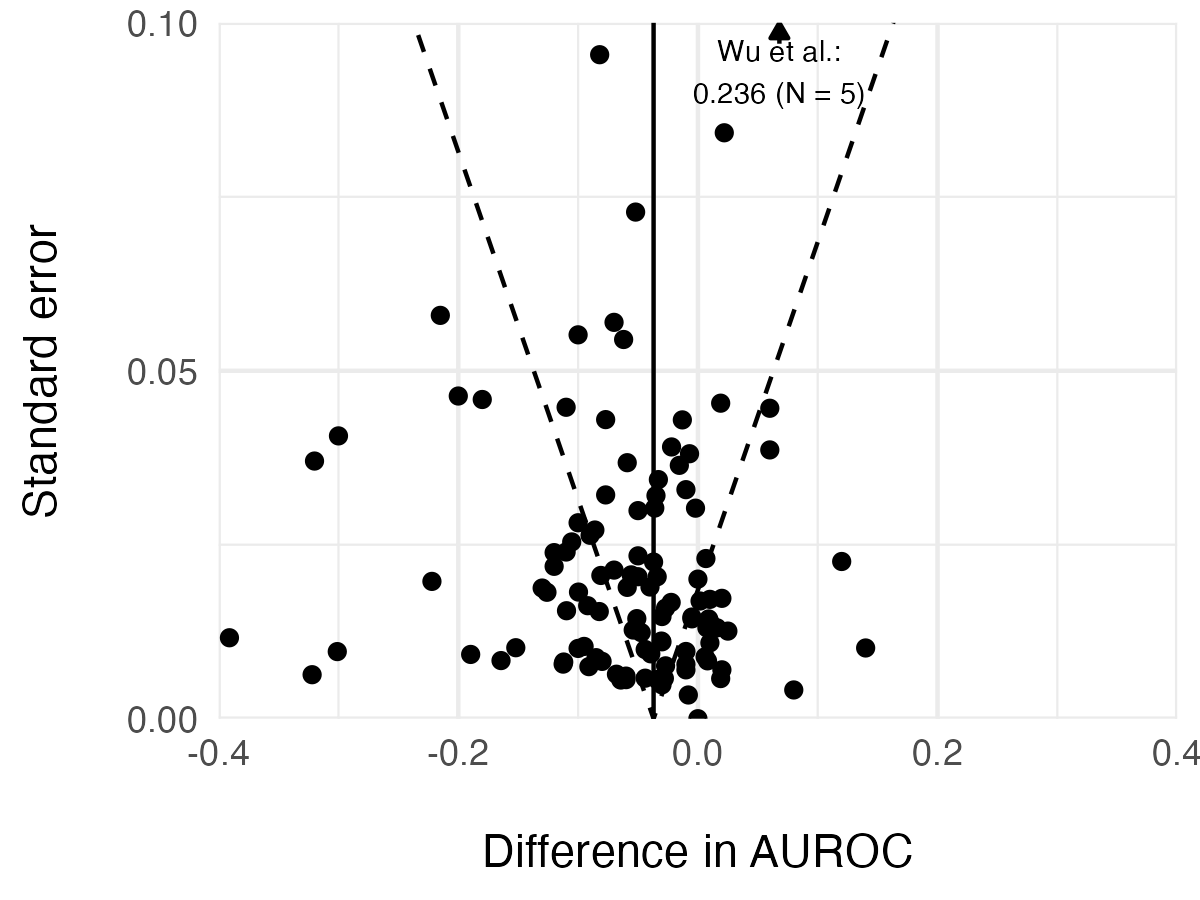
**

**Supplementary Figure 1** - Funnel plot of differences in AUROC between internal and external validation. Standard errors are based on Hanley’s formula, which is a function of sample size, outcome prevalence, and achieved AUROC.

# References

[1. Pirracchio R, Petersen ML, Carone M, Rigon MR, Chevret S, van der Laan MJ. Mortality prediction in intensive care units with the Super ICU Learner Algorithm (SICULA): a population-based study. Lancet Respir Med. 2015;3:42–52.](http://paperpile.com/b/SmPg0w/JUxnd)

[2. Delahanty RJ, Kaufman D, Jones SS. Development and Evaluation of an Automated Machine Learning Algorithm for In-Hospital Mortality Risk Adjustment Among Critical Care Patients. Crit Care Med. 2018;46:e481–8.](http://paperpile.com/b/SmPg0w/FJNMl)

[3. Moon K-J, Jin Y, Jin T, Lee S-M. Development and validation of an automated delirium risk assessment system (Auto-DelRAS) implemented in the electronic health record system. Int J Nurs Stud. 2018;77:46–53.](http://paperpile.com/b/SmPg0w/bSi45)

[4. Huang L, Shea AL, Qian H, Masurkar A, Deng H, Liu D. Patient clustering improves efficiency of federated machine learning to predict mortality and hospital stay time using distributed electronic medical records. J Biomed Inform. 2019;99:103291.](http://paperpile.com/b/SmPg0w/Qk03K)

[5. Liu R, Greenstein JL, Granite SJ, Fackler JC, Bembea MM, Sarma SV, et al. Data-driven discovery of a novel sepsis pre-shock state predicts impending septic shock in the ICU. Sci Rep. 2019;9:6145.](http://paperpile.com/b/SmPg0w/VeWLP)

[6. Shickel B, Loftus TJ, Adhikari L, Ozrazgat-Baslanti T, Bihorac A, Rashidi P. DeepSOFA: A Continuous Acuity Score for Critically Ill Patients using Clinically Interpretable Deep Learning. Sci Rep. 2019;9:1879.](http://paperpile.com/b/SmPg0w/IZM9Y)

[7. van Wyk F, Khojandi A, Kamaleswaran R. Improving Prediction Performance Using Hierarchical Analysis of Real-Time Data: A Sepsis Case Study. IEEE J Biomed Health Inform. 2019;23:978–86.](http://paperpile.com/b/SmPg0w/sOF9a)

[8. Nielsen AB, Thorsen-Meyer H-C, Belling K, Nielsen AP, Thomas CE, Chmura PJ, et al. Survival prediction in intensive-care units based on aggregation of long-term disease history and acute physiology: a retrospective study of the Danish National Patient Registry and electronic patient records. Lancet Digit Health. 2019;1:e78–89.](http://paperpile.com/b/SmPg0w/zhudH)

[9. Kang Y, Jia X, Wang K, Hu Y, Guo J, Cong L, et al. A Clinically Practical and Interpretable Deep Model for ICU Mortality Prediction with External Validation. AMIA Annu Symp Proc. 2020;2020:629–37.](http://paperpile.com/b/SmPg0w/yBcLH)

[10. Zhao Q-Y, Liu L-P, Luo J-C, Luo Y-W, Wang H, Zhang Y-J, et al. A Machine-Learning Approach for Dynamic Prediction of Sepsis-Induced Coagulopathy in Critically Ill Patients With Sepsis. Front Med. 2020;7:637434.](http://paperpile.com/b/SmPg0w/ePSdx)

[11. Roimi M, Neuberger A, Shrot A, Paul M, Geffen Y, Bar-Lavie Y. Early diagnosis of bloodstream infections in the intensive care unit using machine-learning algorithms. Intensive Care Med. 2020;46:454–62.](http://paperpile.com/b/SmPg0w/Eqj9R)

[12. Hyland SL, Faltys M, Hüser M, Lyu X, Gumbsch T, Esteban C, et al. Early prediction of circulatory failure in the intensive care unit using machine learning. Nat Med. 2020;26:364–73.](http://paperpile.com/b/SmPg0w/SZeNB)

[13. Reyna MA, Josef CS, Jeter R, Shashikumar SP, Westover MB, Nemati S, et al. Early Prediction of Sepsis From Clinical Data: The PhysioNet/Computing in Cardiology Challenge 2019. Crit Care Med. 2020;28:210–7.](http://paperpile.com/b/SmPg0w/aaEAf)

[14. Wang Y, Wei Y, Yang H, Li J, Zhou Y, Wu Q. Utilizing imbalanced electronic health records to predict acute kidney injury by ensemble learning and time series model. BMC Med Inform Decis Mak. 2020;20:238.](http://paperpile.com/b/SmPg0w/U4qzy)

[15. Liu X, Hu P, Mao Z, Kuo P-C, Li P, Liu C, et al. Interpretable Machine Learning Model for Early Prediction of Mortality in Elderly Patients with Multiple Organ Dysfunction Syndrome (MODS): a Multicenter Retrospective Study and Cross Validation. arXiv [physics.med-ph]. 2020.](http://paperpile.com/b/SmPg0w/peoPe)

[16. Zhou H, Cheng C, Lipton ZC, Chen GH, Weiss JC. Predicting Mortality Risk in Viral and Unspecified Pneumonia to Assist Clinicians with COVID-19 ECMO Planning. arXiv [stat.AP]. 2020.](http://paperpile.com/b/SmPg0w/gSFDD)

[17. Rahman A, Chang Y, Dong J, Conroy B, Natarajan A, Kinoshita T, et al. Early prediction of hemodynamic interventions in the intensive care unit using machine learning. Crit Care. 2021;25:388.](http://paperpile.com/b/SmPg0w/NJXla)

[18. Zhi D, Zhang M, Lin J, Liu P, Wang Y, Duan M. Establishment and validation of the predictive model for the in-hospital death in patients with sepsis. Am J Infect Control. 2021;49:1515–21.](http://paperpile.com/b/SmPg0w/rTofq)

[19. Holder AL, Shashikumar SP, Wardi G, Buchman TG, Nemati S. A Locally Optimized Data-Driven Tool to Predict Sepsis-Associated Vasopressor Use in the ICU. Crit Care Med. 2021;49:e1196–205.](http://paperpile.com/b/SmPg0w/fZF71)

[20. Hur S, Ko R-E, Yoo J, Ha J, Cha WC, Chung CR. A Machine Learning-Based Algorithm for the Prediction of Intensive Care Unit Delirium (PRIDE): Retrospective Study. JMIR Med Inform. 2021;9:e23401.](http://paperpile.com/b/SmPg0w/LuXki)

[21. Chen Z, Chen M, Sun X, Guo X, Li Q, Huang Y, et al. Analysis of the Impact of Medical Features and Risk Prediction of Acute Kidney Injury for Critical Patients Using Temporal Electronic Health Record Data With Attention-Based Neural Network. Front Med. 2021;8:658665.](http://paperpile.com/b/SmPg0w/Oh6rk)

[22. He J, Lin J, Duan M. Application of Machine Learning to Predict Acute Kidney Disease in Patients With Sepsis Associated Acute Kidney Injury. Front Med. 2021;8:792974.](http://paperpile.com/b/SmPg0w/dHWb9)

[23. Shashikumar SP, Wardi G, Malhotra A, Nemati S. Artificial intelligence sepsis prediction algorithm learns to say “I don’t know.” NPJ Digit Med. 2021;4:134.](http://paperpile.com/b/SmPg0w/47C1I)

[24. Levi R, Carli F, Arévalo AR, Altinel Y, Stein DJ, Naldini MM, et al. Artificial intelligence-based prediction of transfusion in the intensive care unit in patients with gastrointestinal bleeding. BMJ Health Care Inform. 2021;28.](http://paperpile.com/b/SmPg0w/KFvTX)

[25. Ding C, Hu T. Development and External Verification of a Nomogram for Patients with Persistent Acute Kidney Injury in the Intensive Care Unit. Int J Gen Med. 2021;14:5005–15.](http://paperpile.com/b/SmPg0w/kfzwj)

[26. Huang H, Liu Y, Wu M, Gao Y, Yu X. Development and validation of a risk stratification model for predicting the mortality of acute kidney injury in critical care patients. Ann Transl Med. 2021;9:323.](http://paperpile.com/b/SmPg0w/Bik2z)

[27. Singhal L, Garg Y, Yang P, Tabaie A, Wong AI, Mohammed A, et al. eARDS: A multi-center validation of an interpretable machine learning algorithm of early onset Acute Respiratory Distress Syndrome (ARDS) among critically ill adults with COVID-19. PLoS One. 2021;16:e0257056.](http://paperpile.com/b/SmPg0w/AJ8dZ)

[28. Sung M, Hahn S, Han CH, Lee JM, Lee J, Yoo J, et al. Event Prediction Model Considering Time and Input Error Using Electronic Medical Records in the Intensive Care Unit: Retrospective Study. JMIR Med Inform. 2021;9:e26426.](http://paperpile.com/b/SmPg0w/jEVPG)

[29. Liu Z, Khojandi A, Mohammed A, Li X, Chinthala LK, Davis RL, et al. HeMA: A hierarchically enriched machine learning approach for managing false alarms in real time: A sepsis prediction case study. Comput Biol Med. 2021;131:104255.](http://paperpile.com/b/SmPg0w/IQd11)

[30. Shawwa K, Ghosh E, Lanius S, Schwager E, Eshelman L, Kashani KB. Predicting acute kidney injury in critically ill patients using comorbid conditions utilizing machine learning. Clin Kidney J. 2021;14:1428–35.](http://paperpile.com/b/SmPg0w/K2qw8)

[31. Mamandipoor B, Yeung W, Agha-Mir-Salim L, Stone DJ, Osmani V, Celi LA. Prediction of blood lactate values in critically ill patients: a retrospective multi-center cohort study. J Clin Monit Comput. 2022;36:1087–97.](http://paperpile.com/b/SmPg0w/5j1YE)

[32. Moor M, Bennet N, Plecko D, Horn M, Rieck B, Meinshausen N, et al. Predicting sepsis in multi-site, multi-national intensive care cohorts using deep learning. arXiv [cs.LG]. 2021.](http://paperpile.com/b/SmPg0w/liFQe)

[33. Peng X, Li L, Wang X, Zhang H. A Machine Learning-Based Prediction Model for Acute Kidney Injury in Patients With Congestive Heart Failure. Front Cardiovasc Med. 2022;9:842873.](http://paperpile.com/b/SmPg0w/lzFYg)

[34. Luo C, Zhu Y, Zhu Z, Li R, Chen G, Wang Z. A machine learning-based risk stratification tool for in-hospital mortality of intensive care unit patients with heart failure. J Transl Med. 2022;20:136.](http://paperpile.com/b/SmPg0w/IvBY1)

[35. Kim HB, Nguyen HT, Jin Q, Tamby S, Gelaf Romer T, Sung E, et al. Computational signatures for post-cardiac arrest trajectory prediction: Importance of early physiological time series. Anaesth Crit Care Pain Med. 2022;41:101015.](http://paperpile.com/b/SmPg0w/mgnH6)

[36. Fu S, Wang Q, Chen W, Liu H, Li H. Development and External Validation of a Nomogram for Predicting Acute Kidney Injury in Cardiogenic Shock Patients in Intensive Care Unit. Int J Gen Med. 2022;15:3965–75.](http://paperpile.com/b/SmPg0w/s0TsG)

[37. Zhang X, Chen S, Lai K, Chen Z, Wan J, Xu Y. Machine learning for the prediction of acute kidney injury in critical care patients with acute cerebrovascular disease. Ren Fail. 2022;44:43–53.](http://paperpile.com/b/SmPg0w/8IdMW)

[38. Jiang X, Wang Y, Pan Y, Zhang W. Prediction Models for Sepsis-Associated Thrombocytopenia Risk in Intensive Care Units Based on a Machine Learning Algorithm. Front Med. 2022;9:837382.](http://paperpile.com/b/SmPg0w/wGbal)

[39. Liang Q, Xu Y, Zhou Y, Chen X, Chen J, Huang M. Severe acute kidney injury predicting model based on transcontinental databases: a single-centre prospective study. BMJ Open. 2022;12:e054092.](http://paperpile.com/b/SmPg0w/URq6N)

[40. Sharafutdinov K, Bhat JS, Fritsch SJ, Nikulina K, E Samadi M, Polzin R, et al. Application of convex hull analysis for the evaluation of data heterogeneity between patient populations of different origin and implications of hospital bias in downstream machine-learning-based data processing: A comparison of 4 critical-care patient datasets. Front Big Data. 2022;5:603429.](http://paperpile.com/b/SmPg0w/c9IdC)

[41. Chang H-H, Chiang J-H, Wang C-S, Chiu P-F, Abdel-Kader K, Chen H, et al. Predicting Mortality Using Machine Learning Algorithms in Patients Who Require Renal Replacement Therapy in the Critical Care Unit. J Clin Med Res. 2022;11.](http://paperpile.com/b/SmPg0w/KW0cg)

[42. Bai Y, Xia J, Huang X, Chen S, Zhan Q. Using machine learning for the early prediction of sepsis-associated ARDS in the ICU and identification of clinical phenotypes with differential responses to treatment. Front Physiol. 2022;13:1050849.](http://paperpile.com/b/SmPg0w/9bIJP)

[43. Kang S, Park C, Lee J, Yoon D. Machine Learning Model for the Prediction of Hemorrhage in Intensive Care Units. Healthc Inform Res. 2022;28:364–75.](http://paperpile.com/b/SmPg0w/FeXqK)

[44. Rangan ES, Pathinarupothi RK, Anand KJS, Snyder MP. Performance effectiveness of vital parameter combinations for early warning of sepsis-an exhaustive study using machine learning. JAMIA Open. 2022;5:ooac080.](http://paperpile.com/b/SmPg0w/SiWHy)

[45. Luo X-Q, Yan P, Duan S-B, Kang Y-X, Deng Y-H, Liu Q, et al. Development and Validation of Machine Learning Models for Real-Time Mortality Prediction in Critically Ill Patients With Sepsis-Associated Acute Kidney Injury. Front Med. 2022;9:853102.](http://paperpile.com/b/SmPg0w/gJLEv)

[46. Chen Q, Li R, Lin C, Lai C, Chen D, Qu H, et al. Transferability and interpretability of the sepsis prediction models in the intensive care unit. BMC Med Inform Decis Mak. 2022;22:343.](http://paperpile.com/b/SmPg0w/G0hNq)

[47. Alfieri F, Ancona A, Tripepi G, Randazzo V, Paviglianiti A, Pasero E, et al. External validation of a deep-learning model to predict severe acute kidney injury based on urine output changes in critically ill patients. J Nephrol. 2022;35:2047–56.](http://paperpile.com/b/SmPg0w/GesrY)

[48. Choi MH, Kim D, Choi EJ, Jung YJ, Choi YJ, Cho JH, et al. Mortality prediction of patients in intensive care units using machine learning algorithms based on electronic health records. Sci Rep. 2022;12:7180.](http://paperpile.com/b/SmPg0w/yFJ9k)

[49. Yang B, Zhu Y, Lu X, Shen C. A Novel Composite Indicator of Predicting Mortality Risk for Heart Failure Patients With Diabetes Admitted to Intensive Care Unit Based on Machine Learning. Front Endocrinol . 2022;13:917838.](http://paperpile.com/b/SmPg0w/uuvzt)

[50. Dung-Hung C, Cong T, Zeyu J, Yu-Shan O-Y, Yung-Yan L. External validation of a machine learning model to predict hemodynamic instability in intensive care unit. Crit Care. 2022;26:215.](http://paperpile.com/b/SmPg0w/Ttx8r)

[51. Mirzakhani F, Sadoughi F, Hatami M, Amirabadizadeh A. Which model is superior in predicting ICU survival: artificial intelligence versus conventional approaches. BMC Med Inform Decis Mak. 2022;22:167.](http://paperpile.com/b/SmPg0w/VTf3q)

[52. Wu J, Liu C, Xie L, Li X, Xiao K, Xie G, et al. Early prediction of moderate-to-severe condition of inhalation-induced acute respiratory distress syndrome via interpretable machine learning. BMC Pulm Med. 2022;22:193.](http://paperpile.com/b/SmPg0w/rFln1)

[53. Rockenschaub P, Hilbert A, Kossen T, Elbers P, von Dincklage F, Madai VI, et al. The Impact of Multi-Institution Datasets on the Generalizability of Machine Learning Prediction Models in the ICU. Crit Care Med. 2024. https://doi.org/](http://paperpile.com/b/SmPg0w/9lSvh)[10.1097/CCM.0000000000006359](http://dx.doi.org/10.1097/CCM.0000000000006359)[.](http://paperpile.com/b/SmPg0w/9lSvh)

[54. Mesinovic M, Watkinson P, Zhu T. XMI-ICU: Explainable Machine Learning Model for Pseudo-Dynamic Prediction of Mortality in the ICU for Heart Attack Patients. arXiv [cs.LG]. 2023.](http://paperpile.com/b/SmPg0w/BKWtg)

[55. Contreras M, Silva B, Shickel B, Ozrazgat-Baslanti T, Ren Y, Guan Z, et al. APRICOT-Mamba: Acuity Prediction in Intensive Care Unit (ICU): Development and Validation of a Stability, Transitions, and Life-Sustaining Therapies Prediction Model. arXiv [cs.AI]. 2023.](http://paperpile.com/b/SmPg0w/zTXVd)

[56. Zhu CQ, Tian M, Semenova L, Liu J, Xu J, Scarpa J, et al. Fast and Interpretable Mortality Risk Scores for Critical Care Patients. arXiv [cs.LG]. 2023.](http://paperpile.com/b/SmPg0w/J307m)

[57. Zhang S, Duan Y, Hou F, Yan G, Li S, Wang H, et al. Early prediction of sepsis using a high-order Markov dynamic Bayesian network (HMDBN) classifier. Applied Intelligence. 2023;53:26384–99.](http://paperpile.com/b/SmPg0w/2Rtp8)

[58. Liu X, Hu P, Yeung W, Zhang Z, Ho V, Liu C, et al. Illness severity assessment of older adults in critical illness using machine learning (ELDER-ICU): an international multicentre study with subgroup bias evaluation. Lancet Digit Health. 2023;5:e657–67.](http://paperpile.com/b/SmPg0w/CJlSB)

[59. Ren Y, Zhang Y, Zhan J, Sun J, Luo J, Liao W, et al. Machine learning for prediction of delirium in patients with extensive burns after surgery. CNS Neurosci Ther. 2023;29:2986–97.](http://paperpile.com/b/SmPg0w/j9geg)

[60. Wang B, Li Y, Tian Y, Ju C, Xu X, Pei S. Novel pneumonia score based on a machine learning model for predicting mortality in pneumonia patients on admission to the intensive care unit. Respir Med. 2023;217:107363.](http://paperpile.com/b/SmPg0w/Ze31B)

[61. Alfieri F, Ancona A, Tripepi G, Rubeis A, Arjoldi N, Finazzi S, et al. Continuous and early prediction of future moderate and severe Acute Kidney Injury in critically ill patients: Development and multi-centric, multi-national external validation of a machine-learning model. PLoS One. 2023;18:e0287398.](http://paperpile.com/b/SmPg0w/MnmHf)

[62. Shi H, Shen Y, Li L. Early prediction of acute kidney injury in patients with gastrointestinal bleeding admitted to the intensive care unit based on extreme gradient boosting. Front Med. 2023;10:1221602.](http://paperpile.com/b/SmPg0w/OiimZ)

[63. Huang C-T, Wang T-J, Kuo L-K, Tsai M-J, Cia C-T, Chiang D-H, et al. Federated machine learning for predicting acute kidney injury in critically ill patients: a multicenter study in Taiwan. Health Inf Sci Syst. 2023;11:48.](http://paperpile.com/b/SmPg0w/5KJZE)

[64. Zeng Z, Liu Y, Yao S, Liu J, Xiao B, Liu C, et al. Neural networks based on attention architecture are robust to data missingness for early predicting hospital mortality in intensive care unit patients. Digit Health. 2023;9:20552076231171482.](http://paperpile.com/b/SmPg0w/Vcm6q)

[65. Zheng L, Lin Y, Fang K, Wu J, Zheng M. Derivation and validation of a risk score to predict acute kidney injury in critically ill cirrhotic patients. Hepatol Res. 2023;53:701–12.](http://paperpile.com/b/SmPg0w/7cBjM)

[66. Ren W, Zou K, Huang S, Xu H, Zhang W, Shi X, et al. Prediction of in-hospital Mortality of Intensive Care Unit Patients with Acute Pancreatitis Based on an Explainable Machine Learning Algorithm. J Clin Gastroenterol. 2024;58:619–26.](http://paperpile.com/b/SmPg0w/nRUbU)

[67. Ma X, Wang M, Lin S, Zhang Y, Zhang Y, Ouyang W, et al. Knowledge and data-driven prediction of organ failure in critical care patients. Health Inf Sci Syst. 2023;11:7.](http://paperpile.com/b/SmPg0w/tijgM)

[68. Fan Z, Jiang J, Xiao C, Chen Y, Xia Q, Wang J, et al. Construction and validation of prognostic models in critically Ill patients with sepsis-associated acute kidney injury: interpretable machine learning approach. J Transl Med. 2023;21:406.](http://paperpile.com/b/SmPg0w/kyZvg)

[69. Yamga E, Mantena S, Rosen D, Bucholz EM, Yeh RW, Celi LA, et al. Optimized Risk Score to Predict Mortality in Patients With Cardiogenic Shock in the Cardiac Intensive Care Unit. J Am Heart Assoc. 2023;12:e029232.](http://paperpile.com/b/SmPg0w/JxbFv)

[70. Neyra JA, Ortiz-Soriano V, Liu LJ, Smith TD, Li X, Xie D, et al. Prediction of Mortality and Major Adverse Kidney Events in Critically Ill Patients With Acute Kidney Injury. Am J Kidney Dis. 2023;81:36–47.](http://paperpile.com/b/SmPg0w/nmpiy)

[71. Zheng L, Lu Y, Wu J, Zheng M. Development and validation of a prognostic nomogram model for ICU patients with alcohol-associated cirrhosis. Dig Liver Dis. 2023;55:498–504.](http://paperpile.com/b/SmPg0w/22kRw)

[72. Bao C, Deng F, Zhao S. Machine-learning models for prediction of sepsis patients mortality. Med Intensiva. 2023;47:315–25.](http://paperpile.com/b/SmPg0w/5xAYe)

[73. Huang S, Teng Y, Du J, Zhou X, Duan F, Feng C. Internal and external validation of machine learning-assisted prediction models for mechanical ventilation-associated severe acute kidney injury. Aust Crit Care. 2023;36:604–12.](http://paperpile.com/b/SmPg0w/27maF)

[74. Verhaeghe J, De Corte T, Sauer CM, Hendriks T, Thijssens OWM, Ongenae F, et al. Generalizable calibrated machine learning models for real-time atrial fibrillation risk prediction in ICU patients. Int J Med Inform. 2023;175:105086.](http://paperpile.com/b/SmPg0w/f2Pyy)

[75. Liao W, Voldman J. A Multidatabase ExTRaction PipEline (METRE) for facile cross validation in critical care research. J Biomed Inform. 2023;141:104356.](http://paperpile.com/b/SmPg0w/hHmqo)

[76. Xie W, Li Y, Meng X, Zhao M. Machine learning prediction models and nomogram to predict the risk of in-hospital death for severe DKA: A clinical study based on MIMIC-IV, eICU databases, and a college hospital ICU. Int J Med Inform. 2023;174:105049.](http://paperpile.com/b/SmPg0w/A9QwD)

[77. Zhou H, Liu L, Zhao Q, Jin X, Peng Z, Wang W, et al. Machine learning for the prediction of all-cause mortality in patients with sepsis-associated acute kidney injury during hospitalization. Front Immunol. 2023;14:1140755.](http://paperpile.com/b/SmPg0w/G7oVs)

[78. Ishii E, Nawa N, Hashimoto S, Shigemitsu H, Fujiwara T. Development, validation, and feature extraction of a deep learning model predicting in-hospital mortality using Japan’s largest national ICU database: a validation framework for transparent clinical Artificial Intelligence (cAI) development. Anaesthesia Critical Care & Pain Medicine. 2023;42:101167.](http://paperpile.com/b/SmPg0w/H2iOw)

[79. Huang T, Le D, Yuan L, Xu S, Peng X. Machine learning for prediction of in-hospital mortality in lung cancer patients admitted to intensive care unit. PLoS One. 2023;18:e0280606.](http://paperpile.com/b/SmPg0w/DfL7J)

[80. Ouyang Y, Cheng M, He B, Zhang F, Ouyang W, Zhao J, et al. Interpretable machine learning models for predicting in-hospital death in patients in the intensive care unit with cerebral infarction. Comput Methods Programs Biomed. 2023;231:107431.](http://paperpile.com/b/SmPg0w/1S5cx)

[81. Lei M, Han Z, Wang S, Han T, Fang S, Lin F, et al. A machine learning-based prediction model for in-hospital mortality among critically ill patients with hip fracture: An internal and external validated study. Injury. 2023;54:636–44.](http://paperpile.com/b/SmPg0w/Ojh8X)

[82. Ye Z, An S, Gao Y, Xie E, Zhao X, Guo Z, et al. The prediction of in-hospital mortality in chronic kidney disease patients with coronary artery disease using machine learning models. Eur J Med Res. 2023;28:33.](http://paperpile.com/b/SmPg0w/qxA5r)

[83. Zhuang J, Huang H, Jiang S, Liang J, Liu Y, Yu X. A generalizable and interpretable model for mortality risk stratification of sepsis patients in intensive care unit. BMC Med Inform Decis Mak. 2023;23:185.](http://paperpile.com/b/SmPg0w/sFyRD)

[84. Zhang Y, Hu J, Hua T, Zhang J, Zhang Z, Yang M. Development of a machine learning-based prediction model for sepsis-associated delirium in the intensive care unit. Sci Rep. 2023;13:12697.](http://paperpile.com/b/SmPg0w/NSPwM)
